# Multi-level Factors Associated with HIV Late Presentation with Advanced Disease and Delay Time of Diagnosis in South Carolina, 2005–2019

**DOI:** 10.1007/s10461-024-04414-y

**Published:** 2024-06-19

**Authors:** Fanghui Shi, Jiajia Zhang, Shujie Chen, Xueying Yang, Zhenlong Li, Sharon Weissman, Bankole Olatosi, Xiaoming Li

**Affiliations:** 1 Arnold School of Public Health, South Carolina SmartState Center for Healthcare Quality, University of South Carolina, Columbia, SC 29208 USA; 2 SC SmartState Center for Healthcare Quality (CHQ), Department of Health Promotion, Education and Behavior, Arnold School of Public Health, University of South Carolina, 915 Greene Street, Columbia, SC 29208 USA; 3 Department of Epidemiology and Biostatistics, Arnold School of Public Health, University of South Carolina, Columbia, SC 29208 USA; 4 Department of Geography, College of Earth and Mineral Sciences, The Pennsylvania State University, University Park, PA 16802 USA; 5 Department of Internal Medicine, School of Medicine, University of South Carolina, Columbia, SC 29208 USA; 6 Department of Health Services Policy and Management, Arnold School of Public Health, University of South Carolina, Columbia, SC 29208 USA

**Keywords:** HIV, AIDS, Late presentation, Delay time, Electronic health records

## Abstract

This study explored individual- and county-level risk factors of late presentation with advanced disease (LPAD) among people with HIV (PWH) and their longer delay time from infection to diagnosis in South Carolina (SC), using SC statewide Enhanced HIV/AIDS Reporting System (eHARS). LPAD was defined as having an AIDS diagnosis within three months of initial HIV diagnosis, and delay time from HIV infection to diagnosis was estimated using CD4 depletion model. 3,733 (41.88%) out of 8,913 adult PWH diagnosed from 2005 to 2019 in SC were LPAD, and the median delay time was 13.04 years. Based on the generalized estimating equations models, PWH who were male (adjusted prevalence ratio [aPR]: 1.22, 95% CI: 1.12 ∼ 1.33), aged 55+ (aPR: 1.76, 95% CI: 1.62 ∼ 1.92), were Black (aPR: 1.09, 95% CI: 1.03 ∼ 1.15) or Hispanic (aPR: 1.42, 95% CI: 1.26 ∼ 1.61), and living in counties with a larger proportion of unemployment individuals (aPR: 1.02, 95% CI: 1.01 ∼ 1.03) were more likely to be LPAD. Among PWH who were LPAD, Hispanic (adjusted beta: 1.17, 95% CI: 0.49 ∼ 1.85) instead of Black (adjusted beta: 0.11, 95% CI: -0.30 ∼ 0.52) individuals had significant longer delay time compared to White individuals. Targeted and sustained interventions are needed for older, male, Hispanic or Black individuals and those living in counties with a higher percentage of unemployment because of their higher risk of LPAD. Additionally, specific attention should be paid to Hispanic individuals due to their longer delay time to diagnosis.

**Supplementary Information:**

The online version contains supplementary material available at 10.1007/s10461-024-04414-y.

## Introduction

Late HIV presentation is a core indicator for monitoring the progress of HIV testing interventions funded by the U.S. Department of Health and Human Services [1] and one indicator for monitoring national health in Healthy People 2030 [2]. Individuals who are not aware of their HIV status at an early stage of infection cannot benefit from prompt linkage to medical care and antiretroviral treatment, and late presentation with advanced HIV disease (“LPAD”) is associated with a greater risk of severe disease and death [3–5]. There were more than 20 different definitions of HIV late presentation, such as the concurrence of HIV and AIDS diagnosis or having AIDS diagnosis within one year of initial HIV diagnosis. The consensus definition was reached in October 2009 in the Europe 2009 Conference at the Nobel Forum in Stockholm, and it was agreed upon that LPAD is defined as persons presenting for care with a CD4 count below 200 cells/ μL or presenting with an AIDS-defining event, regardless of the CD4 cell count [6]. Decreasing late HIV diagnosis has been increasingly emphasized as part of a comprehensive HIV prevention strategy for ending the HIV epidemic [4]. In 2020, 21.6% of new HIV diagnoses in the United States were LPAD, and South Carolina is one of the ten states in the United States with an LPAD percentage higher than 24.7%. Given the high percentage of HIV late presentation in South Carolina, it is imperative to understand the risk factors associated with LPAD to develop targeted interventions and practical HIV testing strategies.

Based on the modified socioecological framework used to understand the risks of the HIV epidemic, late HIV presentation may be driven by multi-level social risk factors [7]. Various structural-level characteristics, such as income inequality, socioeconomic deprivation, and high unemployment rate, are positively associated with late presentation [8, 9]. At the individual level, higher percentages of late presentation were found among persons who were Black or Hispanic/Latino [10, 11], older [12, 13], male [14], and lived in rural areas [10]. However, there are some knowledge gaps. For example, evidence linking HIV transmission mode and late presentation is mixed. According to a literature review, a positive relationship between heterosexual exposure and late presentation was observed in 16 out of 27 studies reviewed [11]. However, some studies found that infection attributed to men who have sex with men (MSM) was a risk factor for late presentation [12]. Additionally, few studies focused on time to engagement in care among people who were diagnosed late, limiting our ability to identify the risk factors of longer delay time among people with late HIV presentation. To know more about the underlying personal, environmental, and physical risk factors, there is a need for large real-world cohort studies to investigate both individual- and neighborhood-level risk factors of late HIV presentation and the length of delay time from infection to diagnosis.

By linking statewide individual-level public health surveillance data and publicly available county-level surveillance datasets (e.g., American Community Survey), this study aims to (1) explore the temporal variation of HIV LPAD and delay time to diagnoses and (2) identify the association of multi-level characteristics (e.g., individual demographic characteristics and county-level economic status) with HIV LPAD and delay time of diagnosis.

## Methods

### Study Population and data Source

All adults (≥ 18 years old) diagnosed with HIV in South Carolina between January 2005 and December 2019 and with at least one CD4 count record or AIDS diagnosis within three months of initial HIV diagnosis were included in this study. Their deidentified information, including HIV/AIDS diagnosis date, demographic characteristics (e.g., age, gender, race/ethnicity, and residence), HIV transmission mode, and laboratory results (e.g., CD4 count and viral load) were extracted from the South Carolina statewide Enhanced HIV/AIDS Reporting System. This public health surveillance database contains all HIV cases reported to the South Carolina Department of Health and Environmental Control. Individual data in the Enhanced HIV/AIDS Reporting System were further linked with county-level publicly available datasets (e.g., County Health Rankings and American community survey) via the Federal Information Procession Standards code, a residential county identifier. For the purpose of data analysis, the current study only included PWH with at least one CD4 cell count record or AIDS diagnosis within three months of initial HIV diagnosis (9,588 out of 11,209, 85.54%) [13]. The institutional review boards at the University of South Carolina and relevant South Carolina state agencies approved the study protocol.

### Outcome

In the current study, LPAD was a binary outcome indicating whether a person has an AIDS diagnosis or lab record of CD4 count < 200 cells/μL within three months of initial HIV diagnosis [6]. To be more specific, a person was considered as “LPAD” if any of the following criteria were met: (1) was diagnosed with AIDS at the time of initial HIV diagnosis; (2) had AIDS diagnosis within three months of initial HIV diagnosis; or (3) had CD4 cell counts record less than 200 cells/μL within three months of initial HIV diagnosis.

Another outcome we were interested in was the delayed time of diagnosis, the estimated interval from HIV infection to initial diagnosis [14, 15]. We estimated the time from HIV infection to diagnosis based on a well-characterized CD4 depletion model commonly used in the literature:

$$\sqrt{CD4\left(t\right)}={a}_{i}+\left({b}_{i}\times t\right)+{e}_{it}$$

Where CD4 is the first CD4 value at HIV diagnosis, t is the duration from HIV infection to the date of the CD4 test, and a_i_ and b_i_ are the specific model parameters to the U.S. HIV population subgroups (sex, transmission category, and age) [16]. The detailed information for CD4 model parameters used for different subsets of populations was described elsewhere [16]. One difficulty in applying this model is the age of infection was unknown. We used the age at the first CD4 test as the approximation of the age at infection to estimate t. The age at infection was then calculated using the date of the first CD4 test minus t. The estimation of t was repeated using the calculated age at infection to determine the final CD4 modeling age group. The diagnosis delay was estimated as follows:

$${\rm{Diagnosis}}\,{\rm{delay}}\,{\rm{ = }}\,{\rm{Date}}\,{\rm{of}}\,{\rm{infection}} - {\rm{Date}}\,{\rm{of}}\,{\rm{first}}\,{\rm{CD4}}\,{\rm{test}}$$

Using data on the first CD4 value after HIV diagnosis during the study period, we calculated the diagnosis delays in PWH with LPAD and with at least one CD4 count record within three months of initial HIV diagnosis.

### Individual-level Factors

Individual-level characteristics were obtained from the Enhanced HIV/AIDS Reporting System and included age at diagnosis (18–34, 35–54, ≥ 55 years old), gender (male, female), and race/ethnicity (Black, White, Hispanic, other/unknown). HIV transmission modes were categorized into heterosexual, MSM, injection drug users (IDU), and other/unknown. Residence information (Federal Information Procession Standards code) was classified into rural and urban areas based on the 2013 USDA Rural-Urban Continuum Codes, with codes 1–3 representing urban residents and 4–9 representing rural residents [17]. The International Classification of Diseases codes for 19 different comorbid disease categories (e.g., congestive heart failure) based on the Charlson Comorbidity Index (CCI) scoring system were extracted to calculate the CCI score [18]. For the updated CCI score, the scores assigned to HIV infection and AIDS were subtracted from the CCI score because all the subjects in the current study were diagnosed with HIV. The total score indicates comorbidity burden, and the higher the score, the higher the burden [19]. The adapted CCI score was further grouped into 0, 1, and ≥ 2 for data analysis.

### County-level Factors

Seven social demographic factors obtained from the American Community Survey five-year estimates included population density, percentage of males, percentage of the population being Black or African American alone, percentage of the total civilian population for whom poverty status is determined, percentage of the civilian labor force that is unemployed, percentage of housing units with no vehicle available, and percentage of the adult population (aged 25 and older) with less than high school education. One healthcare resource-related factor, the number of Ryan White centers within 25 miles radius of each county per 100,000 people, was measured using the geolocation information of Ryan White HIV centers from the United States Department of Health and Human Services. The number of mental health providers per 100,000 residents was another healthcare resource-related factor obtained from the County Health Rankings and Roadmaps Program. The racial dissimilarity index, ranging from 0 to 1 and with higher values indicating greater residential segregation between Black and White residents, was also obtained from County Health Rankings and Roadmaps and used in the analysis.

### Statistical Analysis

The temporal and spatial variations of the annual percentage of LPAD among people diagnosed with HIV from 2005 to 2019 in South Carolina were illustrated by bar plots with a linear regression line and a heat map. The median length of delay time by individual-level characteristics (e.g., residence, age, and gender) across time was illustrated by a bar plot. Generalized estimating equations models with backward selection were built to explore multi-level factors related to LPAD status (binary variable) and delay time (continuous variable). The “GEE” function in SAS with log-binomial and identity links was used for the binary and continuous outcomes, respectively. To capture the repeated value at the county level, the first-order autoregressive structure and compound symmetry correlation matrix was tested in two models, and the results with smaller Quasilikelihood Information Criterion were presented. Detailed SAS code information for the GEE models was provided in supplemental materials.

During the HIV acute infection period, the CD4 count may drop below 200 cells/μL within a few weeks of infection. Thus, we could not differentiate LPAD from those in the acute infection period based only on CD4 cell numbers. One potential way to distinguish the acute and late stages of HIV infection is the serum HIV viral load because the viral load in the acute infection stage often reaches over 1 × 10^6^ copies/mL, which is typically higher than that after several years of infection [20]. To examine the influence of acute infection, we did a sensitivity analysis for delay time by excluding those having CD4 cell counts less than 200 cells/μL and the HIV viral load higher than 1 × 10^6^ copies/mL within three months of initial HIV diagnosis. Statistically significant relationships were considered at *p* < 0.05. Figures were created using R 4.1.3, and all other statistical analyses were conducted in SAS software version 9.4 (SAS Institute, Inc., Cary, NC).

## Results

### Patterns and Temporal Trend for LPAD and Delay Time

The analysis included 8,913 people diagnosed with HIV from 2005 to 2019 in South Carolina. Most were male (75.24%) and Black (69.27%). Of the 8,913 new HIV diagnoses, 3,733 (41.88%) were LPAD. Compared to those who were not LPAD, a greater portion of LPAD were aged ≥ 55 (14.55% vs. 7.72%) and Hispanic (7.85% vs. 4.36%) (Table 1). Among the LPAD, 2,842 had at least one CD4 count record within three months of initial HIV/AIDS diagnosis. Their estimated median delay time from infection to diagnosis was 13.04 years.

**Table 1:** Late presentation with advanced disease (LPAD) by individual and county level factors, South Carolina, 2005–2019

Factors	Overall (*n* = 8,913)	LPAD		
		No (*n* = 5,180)	Yes (*n* = 3,733)	
**Individual-level characteristics** ^a^				
Age (years old)				
18–34	4,535 (50.88)	3,143 (60.68)	1,392 (37.29)	
35–54	3,435 (38.54)	1,637 (31.60)	1,798 (48.17)	
55+	943 (10.58)	400 (7.72)	543 (14.55)	
Gender				
Female	2,207 (24.76)	1,297 (25.04)	910 (24.38)	
Male	6,706 (75.24)	3,883 (74.96)	2,823 (75.62)	
Race/ethnicity				
White	1,999 (22.43)	1,201 (23.19)	798 (21.38)	
Black	6,174 (69.27)	3,612 (69.73)	2,562 (68.63)	
Hispanic	519 (5.82)	226 (4.36)	293 (7.85)	
Other/unknown	221 (2.48)	141 (2.72)	80 (2.14)	
HIV transmission mode				
Heterosexual	1,755 (19.69)	944 (18.22)	811 (21.73)	
MSM	4,486 (50.33)	2,863 (55.27)	1,623 (43.48)	
IDU	481 (5.40)	284 (5.48)	197 (5.28)	
Other	2,191 (24.58)	1,089 (21.02)	1,102 (29.52)	
Residence				
Rural	1,551 (17.40)	870 (16.80)	681 (18.24)	
Urban	7,362 (82.60)	4,310 (83.20)	3,052 (81.76)	
CCI score				
0	6,972 (78.22)	4,078 (78.73)	2,894 (77.52)	
1	1,375 (15.43)	808 (15.60)	567 (15.19)	
>=2	566 (6.35)	294 (5.68)	272 (7.29)	
**County-level characteristics** ^**b**^				
Population density	291.64 (176.14)	299.82 (177.99)	280.29 (172.94)	
% Male	48.51 (1.05)	48.49 (1.03)	48.55 (1.08)	
% Black	32.64 (14.90)	32.78 (14.80)	32.44 (15.03)	
% Poverty	30.06 (18.65)	30.07 (18.26)	30.04 (19.18)	
% Unemployed	9.05 (2.60)	8.95 (2.61)	9.19 (2.58)	
% No access to vehicle	7.43 (2.23)	7.38 (2.19)	7.49 (2.28)	
% Less than high school	15.46 (5.25)	15.10 (5.08)	15.97 (5.42)	
Ryan White centers	1.69 (3.19)	1.62 (3.12)	1.78 (3.29)	
Mental health provider	3.59 (5.56)	3.44 (5.43)	3.81 (5.73)	
Dissimilarity index	0.39 (0.09)	0.39 (0.08)	0.39 (0.09)	

Notes: ^a^ Data are presented as n (%)

^b^ Data are presented as mean (standard deviation)

**Fig. 1:**
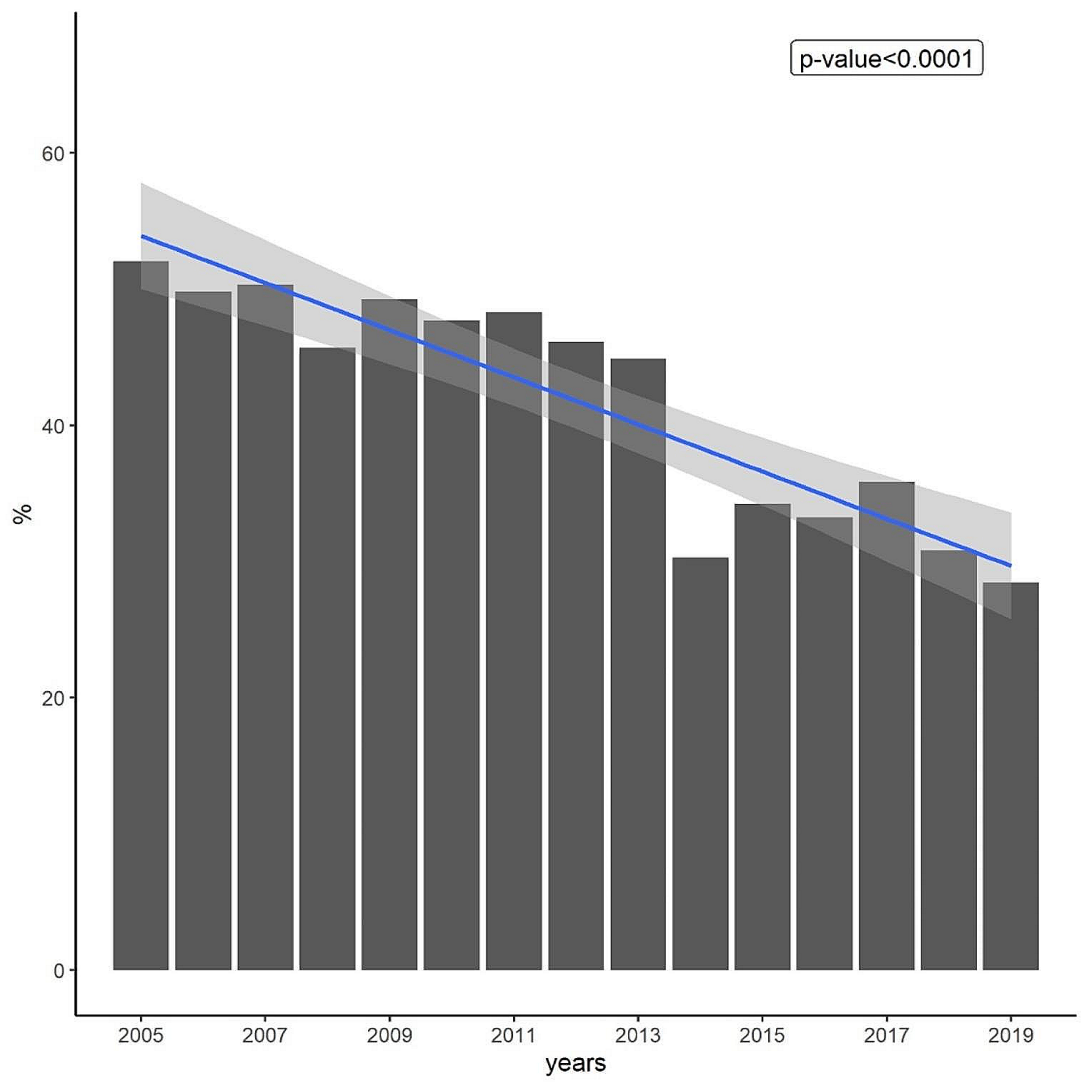
Percent of new HIV diagnosis that were late presentation with advanced HIV disease from 2005 to 2019 in South Carolina: a bar plot with a linear regression line

**Fig. 2:**
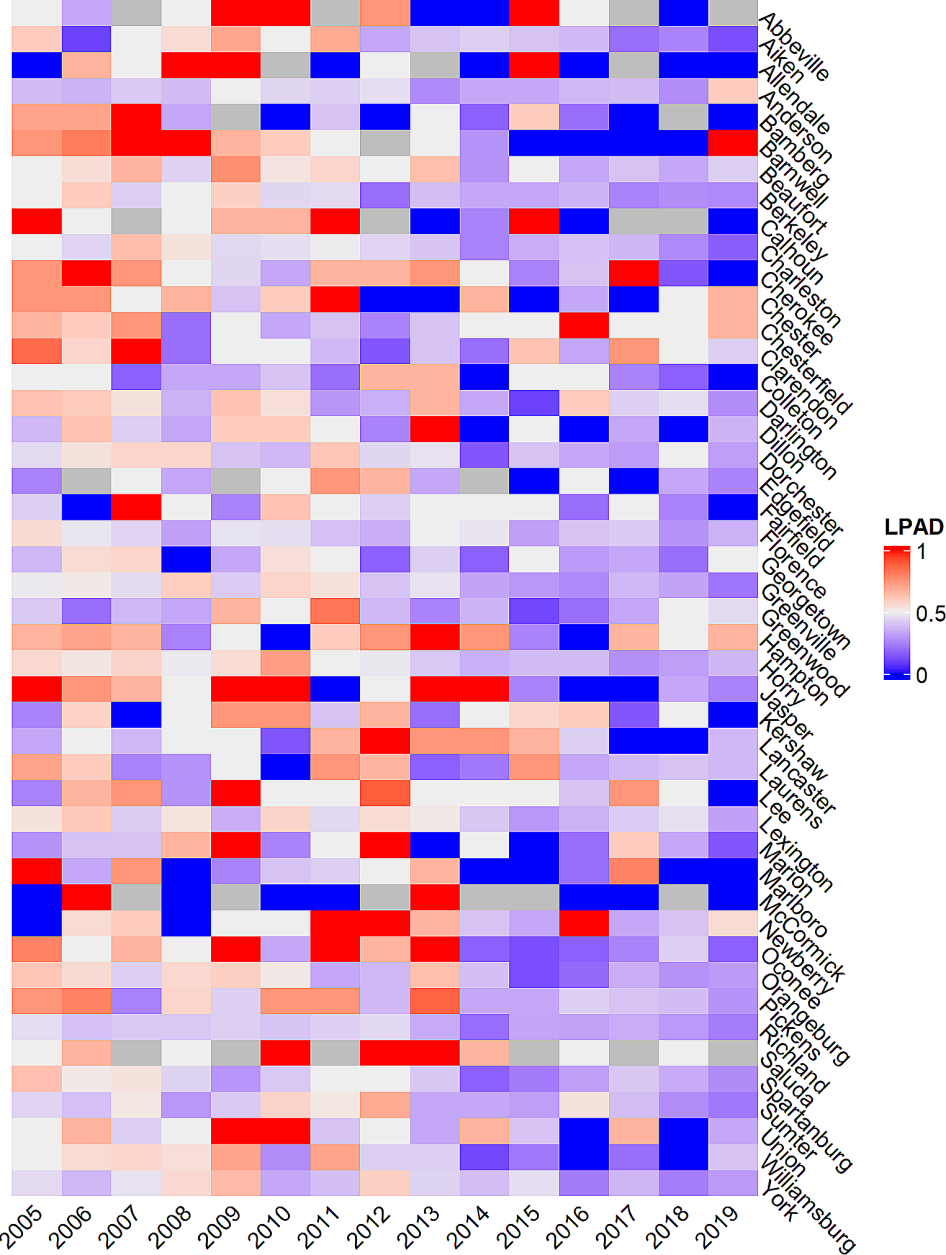
Percent of new HIV diagnosis that were late presentation with advanced disease (LPAD) across 46 counties in South Carolina, 2005–2019

**Fig. 3:**
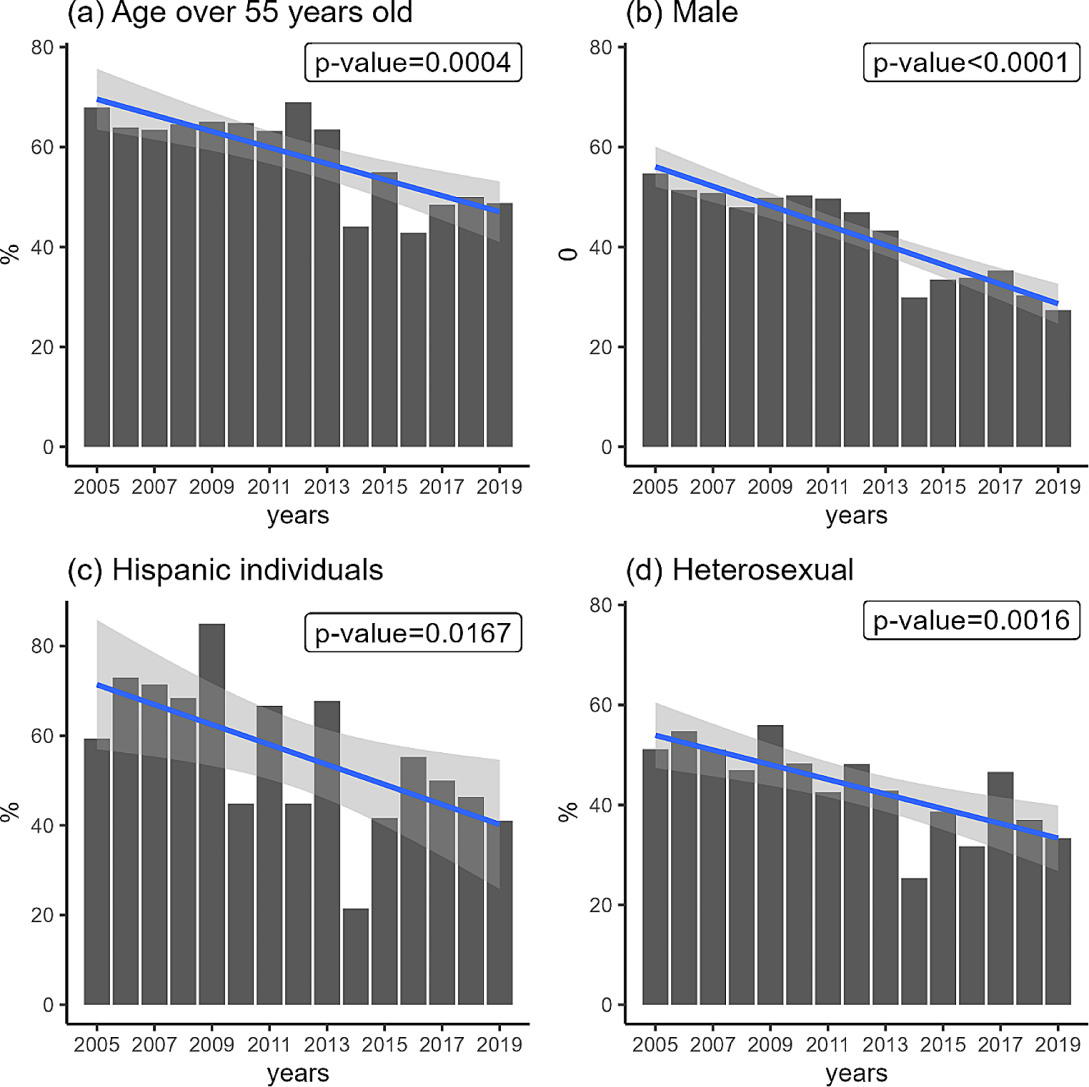
Percent of late presentation with advanced disease among newly diagnosed HIV cases who were aged over 55 years old, male, Hispanic, and with HIV transmission mode being heterosexual: bar plots with linear regression lines

**Fig. 4:**
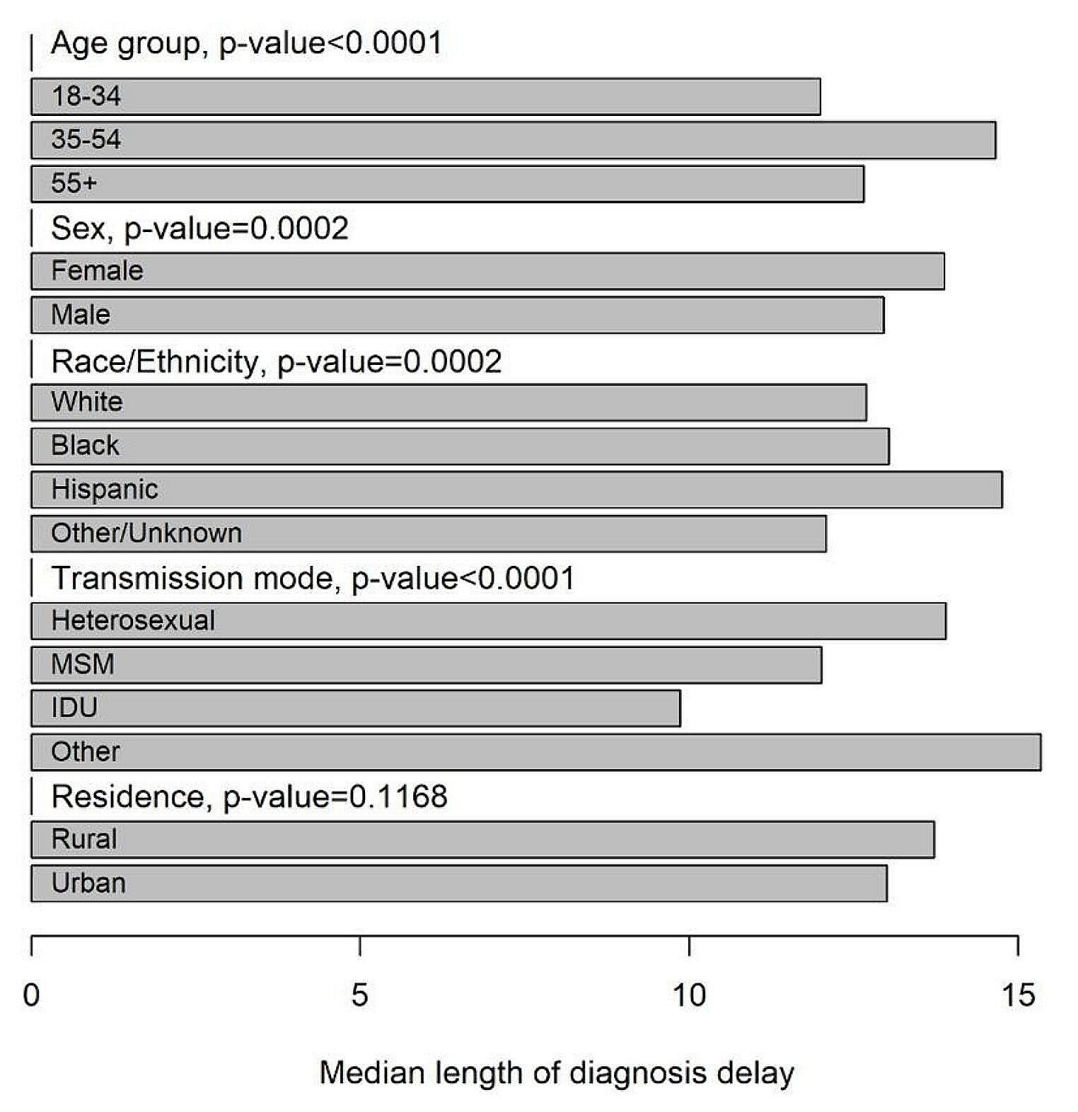
Median length (years) of diagnosis delay among people with late presentation with advanced disease by individual demographic characteristics

As displayed in Fig. 1, there was a significant declining trend from 2005 to 2019 in the percentage of LPAD (52.06% in 2005 and 28.46% in 2019) in South Carolina. When breaking down the annual percentage of LPAD at the county level, an overall declining trend (indicated by fewer red cells in the later phase of Fig. 2) was observed in most counties in South Carolina. As shown in Fig. 3, the declining trend of the percentage of LPAD among newly diagnosed PWH who were aged over 55 years old, male, Hispanic, and with HIV transmission mode being heterosexual from 2005 to 2019 was significant. Delay time to diagnosis was greater among those Hispanic and those aged between 35 and 54 years old. (Fig. 4)

### Factors Associated with LPAD and Delay Time

Compared to PWH aged 18 to 34 years, those aged 35–54 years (adjusted Prevalence ratio [aPR]: 1.57, 95% CI: 1.47 ∼ 1.67) and older than 55 years (aPR: 1.76, 95% CI: 1.62 ∼ 1.92) were more likely to be LPAD. In addition, LPAD were more likely to be male (aPR: 1.22, 95% CI: 1.12 ∼ 1.33) vs. female, and Hispanic (aPR: 1.42, 95% CI: 1.26 ∼ 1.61) vs. White and were more likely to be Black (aPR: 1.08, 95% CI: 1.03 ∼ 1.15) vs. White. Compared to PWH with heterosexual transmission mode, those reporting MSM (aPR: 0.89, 95% CI: 0.82–0.96) and IDU (aPR: 0.87, 95% CI: 0.79–0.96) were less likely to be LPAD. At the county level, PWH living in counties with a higher percentage of unemployment (aPR: 1.02, 95% CI: 1.00 ∼ 1.03) and a lower percentage of Black (aPR: 0.996, 95% CI: 0.993 ∼ 0.999) were more likely to be LPAD. (Table 2)

**Table 2:** Factors associated with late presentation with advanced HIV disease (LPAD): Generalized estimating equations model with backward selection

Factors	Crude PR (95% CI)	Adjusted PR (95% CI)
Age (years old)		
18–34	Ref	Ref
35–54	1.705 (1.615,1.800)***	1.566 (1.465, 1.673)***
55+	1.876 (1.749,2.012)***	1.762 (1.616, 1.921)***
Gender		
Female	Ref	Ref
Male	1.021 (0.964,1.081)	1.222 (1.123, 1.329)***
Race/ethnicity		
White	Ref	Ref
Black	1.039 (0.978,1.105)	1.084 (1.027, 1.145)**
Hispanic	1.414 (1.289,1.552)***	1.421 (1.257, 1.606)***
Other/unknown	0.907 (0.755,1.089)	1.006 (0.816, 1.241)
HIV transmission mode		
Heterosexual	Ref	Ref
MSM	0.783 (0.735,0.834)***	0.888 (0.824, 0.958)**
IDU	0.886 (0.787,0.998)*	0.871 (0.787, 0.963)**
Other	1.088 (1.019,1.162)*	1.064 (1.000, 1.133)
Residence		
Rural	Ref	-
Urban	0.944 (0.887,1.005)	-
CCI score		
0	Ref	-
1	0.993 (0.927,1.064)	-
>=2	1.158 (1.058,1.267)**	-
Diagnosis year	0.960 (0.955,0.966)***	0.969 (0.964, 0.975)***
**County-level characteristics**		
Population density	1.000 (0.999,1.000)***	1.000 (1.000, 1.000)***
% Male	1.033 (0.996,1.071)	-
% Black	1.001 (0.998,1.003)	0.996 (0.995, 0.998)***
% Poverty	0.999 (0.997,1.002)	-
% Unemployed	1.021 (1.009,1.034)***	1.015 (1.005, 1.025)**
% No access to vehicle	1.021 (1.002,1.040)**	-
% Less than high school	1.020 (1.013,1.028)***	-
Ryan White centers	1.006 (0.998,1.014)	-
Mental health provider	1.004 (0.999,1.010)	-
Dissimilarity index	1.027 (0.612,1.724)	-

Notes: - Variables that were removed from the final adjusted model based on backward selection

*p-value < 0.05; **p-value < 0.01; ***p-value < 0.001

PR: Prevalence ratio; MSM: Men who have sex with men; IDU: Injection drug users; CCI: Charlson Comorbidity Index

Similar to the results of LPAD, PWH aged 35–54 years (adjusted beta: 2.18, 95% CI: 1.72 ∼ 2.63) had a longer delay time to diagnosis compared to those aged 18–34 years. Hispanic individuals (adjusted beta: 1.17, 95% CI: 0.49 ∼ 1.85) had a longer delay time, but the association between being Black and delay time disappeared (adjusted beta: 0.11, 95% CI: -0.30 ∼ 0.52). Having the transmission mode as MSM (adjusted beta: -1.39, 95% CI: -2.01 ~ -0.77) or IDU (adjusted beta: -4.03, 95% CI: -4.64 ~ -3.41) was consistently associated with shorter delay time to diagnosis compared to heterosexual transmission. (Table 3) After excluding those having CD4 cell counts less than 200 cells/μL and the first HIV viral load larger than 1 × 10^6^ copies/mL within three months of initial HIV diagnosis (304 out of 2,842 [10.70%]), the sensitivity analysis of delay time shows similar results to the findings in the models with the full sample. (Supplemental Table 1)

**Table 3:** Factors associated with delay time from HIV infection to diagnosis: Generalized estimating equations model with backward selection

Factors	Crude Beta (95% CI)	Adjusted Beta (95% CI)	
Age (years old)			
18–34	Ref	Ref	
35–54	2.503 (2.130, 2.875)***	2.175 (1.716, 2.633)***	
55+	0.379 (-0.132, 0.890)	-0.212 (-0.713, 0.289)	
Gender			
Female	Ref	Ref	
Male	-0.814 (-1.213, -0.415)***	0.278 (-0.255, 0.810)	
Race/ethnicity			
White	Ref	Ref	
Black	0.438 (0.018, 0.859)*	0.109 (-0.298, 0.515)	
Hispanic	1.636 (0.915, 2.357)***	1.169 (0.492, 1.845)***	
Other/unknown	-0.208 (-1.424, 1.009)	0.113 (-0.995, 1.221)	
HIV transmission mode			
Heterosexual	Ref	Ref	
MSM	-1.623 (-2.058, -1.188)***	-1.389 (-2.012, -0.767)***	
IDU	-3.928 (-4.722, -3.134)***	-4.029 (-4.644, -3.414)***	
Other	1.176 (0.716, 1.637)***	1.316 (0.867, 1.765)***	
Residence			
Rural	Ref	Ref	
Urban	-0.414 (-0.857, 0.029)	-0.569 (-1.270, 0.131)	
CCI score			
0	Ref	Ref	
1	0.338 (-0.136, 0.812)	0.237 (-0.173, 0.647)	
>=2	-0.139 (-0.788, 0.509)	-0.367 (-0.837, 0.103)	
Diagnosis year	-0.040 (-0.080, 0.000)	0.969 (0.964, 0.975)***	
County-level characteristics			
Population density	0.999 (0.997, 1.000)	-	
% Male	0.980 (0.766, 1.253)	-	
% Black	0.995 (0.977, 1.013)	-	
% Poverty	0.999 (0.986, 1.012)	-	
% Unemployed	1.010 (0.927, 1.102)	-	
% No access to vehicle	1.034 (0.942, 1.134)	-0.102 (-0.220, 0.016)	
% Less than high school	1.049 (1.004, 1.097)*	0.061 (0.009, 0.113)*	
Ryan White centers	0.991 (0.935, 1.051)	-	
Mental health provider	1.000 (0.955, 1.048)	-0.045 (-0.102, 0.012)	
Dissimilarity index	0.124 (0.011, 1.352)	-	

Notes: - Variables that were removed from the final adjusted model based on backward selection

*p-value < 0.05; **p-value < 0.01; ***p-value < 0.001

MSM: Men who have sex with men; IDU: Injection drug users; CCI: Charlson Comorbidity Index

## Discussion

This study is one of the first attempts to simultaneously explore the association of multi-level factors with LPAD and the length of delay time to diagnosis. Our population-based analysis found that people who were older, male, Black or Hispanic, with the heterosexual HIV transmission mode, and living in counties with a larger proportion of unemployment were more likely to be LPAD. In addition, among people who were LPAD, those who were older, Hispanic, and with heterosexual transmission mode had a longer delay time. Targeted strategies should be tailored to different subgroups to address the barriers to LPAD and delay time.

In this study, our findings regarding the associations of age, gender, race/ethnicity, and HIV transmission mode with LPAD were consistent with most existing literature [11, 16, 21]. Numerous reasons could explain why older adults are more likely to be diagnosed late, such as inaccurate self-perception of risk, failure of medical providers to suggest HIV screening, and medical providers not able to recognize the clinical signs of HIV/AIDS when older adults present with illness [21]. Access to early HIV diagnosis may vary between men and women due to socio-cultural, healthcare access, and economic factors. Some of the women’s healthcare utilization, such as the opt-out test in prenatal care and cervical cancer screening, might contribute to lower likelihood of LPAD among women [22]. Numerous community efforts have been taken to increase HIV testing among gay and bisexual men since the mid-2000s because of their high-risk status [23]. This could partially explain the higher possibility of LPAD in heterosexual PWH. This finding suggests the need for greater public awareness and promotion efforts of HIV testing among this population despite their relatively lower risk of HIV in comparison with key populations such as gay and bisexual men.

Hispanic rather than Black/African American individuals had longer diagnosis delays compared to White individuals. One potential explanation is that except for some common barriers to accessing testing services in Hispanic and Black individuals, such as lack of health insurance, discrimination, and homophobia, Hispanic persons may face additional immigration issues and language barriers. Besides a higher possibility of LPAD, heterosexual persons were also found to have a longer diagnosis delay. This finding is consistent with previous studies [24]. A national-level study based on surveillance data in France found that heterosexual men, whether born in France or abroad, tended to have the longest time intervals from infection to diagnosis [24]. Hispanic and heterosexual persons should be targeted with greater attention to promoting early HIV testing because they are more likely to be LPAD and have longer delay time.

Individuals living in counties with a larger percentage of Black/African Americans were found to be less likely to be LPAD. The percentage of Black/African American residents in each county has been interpreted as a measure of neighborhood racial context, and higher values are postulated to be related to enhanced social cohesion, mutual social support, lower exposure to discrimination, and a sense of community. This could help explain why living in counties with higher percentages of Black residents was related to a lower likelihood of LPAD. CCI scores in the current study did not demonstrate statistically significant associations with LPAD and the duration of diagnosis delay. A potential explanation is that some components in the CCI scoring system (e.g., kidney and liver disease) might be caused by HIV infection, and this could raise temporality concerns when examining the relationship between CCI and HIV late presentation. The absence of significant association between some county-level variables (e.g., the quantity of Ryan White centers, the availability of mental health providers, and the dissimilarity index) and HIV late presentation suggests that these variables were not able to fully capture the complexities of HIV diagnosis patterns and further exploration of other contextual factors related to healthcare access and residential segregation may be needed.

Effective strategies to promote timely HIV testing include but are not limited to learning from the lived experience of PWH with late diagnosis, targeting and removing barriers they face for HIV testing, increasing HIV testing services at community settings, normalizing HIV, reducing HIV-related stigma, and expanding access to at-home HIV self-testing [23]. Literature suggests that self-testing was less stigmatizing for participants. One at-home self-testing program in Canada during the COVID-19 pandemic found that self-testing is one solution to have persons never tested for HIV being tested [25]. Additionally, HIV testing is a requirement for initiating or continuing pre-exposure prophylaxis (PrEP), a medicine that reduces the risk of HIV infection from sex or injecting drug use [26, 27]. Thus, the promotion of PrEP has a direct effect on HIV testing and could also be an effective strategy in reducing later HIV diagnosis. In addition, timely initiation of Highly Active Antiretroviral Therapy (HAART), coupled with direct referral to treatment centers after diagnosis, is pivotal for improving HIV health outcomes among PWH who were LPAD and suppressing onward HIV transmission within communities. Direct referral after diagnosis enables timely access to HIV treatment by mitigating barriers to linkage to care, such as stigma and transportation challenges.

The current study has several limitations that need to be acknowledged. First, the statistics based on the current definition of LPAD might lead to overestimating the number of late-presented patients because of a transient decline in the CD4 count in the acute phase of HIV infection. However, the results of our sensitivity analysis regarding delay time showed similar results to the primary analysis, supporting our findings regarding the risk factors of delay time among people with LPAD [20]. Second, individuals without AIDS diagnosis or CD4 count record within three months of initial HIV diagnosis were excluded from the analysis because of the uncertainty about whether they are LPAD or not. Thus, caution needs to be taken when generalizing findings to all PWH who are LPAD in the South Carolina Enhanced HIV/AIDS Reporting System dataset. Third, we are not able to incorporate some other important individual-level socioeconomic-related characteristics (e.g., insurance status, social support, and education) into our analysis due to the limitation of data availability. These factors may explain some of the differences we observed in LPAD by the proportion of unemployment at the county level. Fourth, caution should be taken when generalizing findings regarding risk factors of delay time. The value of CD4 count is necessary for the calculation of delay time, and those with initial HIV diagnosis being AIDS but having no CD4 counts within three months of initial diagnosis were excluded. Thus, not all people with LPAD were included in the delay time analysis. However, all the excluded late presented PWH have AIDS diagnosis within three months of initial HIV diagnosis, and AIDS diagnosis is based on CD4 count < 200 or opportunistic illness and could indicate linkage to healthcare. Thus, we believe that the excluded population is less likely to be impacted by healthcare-related delays than the included population, and the impact of excluding this population on our findings is minimal. Fifth, the accuracy of diagnosis delay time depends on the precision of the CD4 depletion model, which may undergo alterations over time in response to the virus’s evolving nature. Additionally, age at HIV diagnosis is used as a proxy for age at infection to estimate the duration from HIV infection to the date of the CD4 test, which may influence the accuracy of the CD4 depletion model.

## Conclusion

The percentage of LPAD and the median delay time decreased from 2005 to 2019. Although these results are encouraging, there were consistent and significant demographic and socioeconomic disparities in late HIV diagnosis. Targeted and sustained interventions are needed for older, male, Hispanic, and heterosexual persons due to their higher possibility of LPAD. In addition, specific attention should be paid to people living in rural areas due to their longer delay time. Promoting HIV testing in communities with socioeconomic disadvantages (e.g., counties with a greater proportion of unemployment) with tailored interventions (e.g., expanding at-home HIV self-testing) is warranted to end the HIV epidemic in the South Carolina and beyond.

## Electronic Supplementary Material

Below is the link to the electronic supplementary material.

Supplementary Material 1

## Data Availability

The authors are prohibited from making individual-level data available publicly due to provisions in our data use agreements with state agencies/data providers, institutional policy, and ethical requirements. To facilitate research, we make access to such data available via approved data access requests through the data owners. The data is unavailable externally or for re-release due to prohibitions in data use agreements with our state agencies or other data providers. Institutional policies stipulate that all external requests for data access require collaboration with a USC researcher. For more information or to make a request, please contact (Bankole Olatosi, PhD): Olatosi@mailbox.sc.edu. The underlying analytical codes are available from the authors on request.
