# Supplementary material for: Multi-level Factors Associated with HIV Late Presentation with Advanced Disease and Delay Time of Diagnosis in South Carolina, 2005–2019
Source: AIDS Behav. 2024 Jun 19;28(10):3205–16. doi: 10.1007/s10461-024-04414-y (PMC11427474; doi:10.1007/s10461-024-04414-y)
Supplement: Supplementary file 1 — Supplementary Material 1 [file 10461_2024_4414_MOESM1_ESM.docx]

Table of Contents

[Supplemental Table 1 Sensitivity analysis of factors associated with delay time: Generalized estimating equations model 2](#_Toc167445094)

[Supplemental SAS code: Generalized estimating equations 5](#_Toc167445095)

| Supplemental Table 1 Sensitivity analysis of factors associated with delay time: Generalized estimating equations model | | |  |
| --- | --- | --- | --- |
| **Factors** | **Delay Time (N = 2538)** | | |
|  | **Adjusted Beta**  **(95% CI)** | **p-value** | |
| **Individual-level characteristics** |  |  | |
| Age (years old) |  |  | |
| 18-34 | 0 |  | |
| 35-54 | 2.140 (1.694, 2.586) | <0.0001 | |
| 55+ | -0.248 (-0.770, 0.274) | 0.3526 | |
| Sex |  |  | |
| Female | 0 |  | |
| Male | 0.259 (-0.283, 0.800) | 0.3489 | |
| Race/Ethnicity |  |  | |
| White | 0 | 0 | |
| Black | 0.110 (-0.329, 0.549) | 0.622 | |
| Hispanic | 1.100 (0.339, 1.861) | 0.0046 | |
| Other/unknown | 0.176 (-0.936, 1.287) | 0.7566 | |
| HIV Transmission mode |  |  | |
| Heterosexual | 0 |  | |
| MSM | -1.381 (-2.050, -0.711) | <0.0001 | |
| IDU | -4.001 (-4.632, -3.369) | <0.0001 | |
| Other | 1.305 (0.811, 1.800) | <0.0001 | |
| Residence |  |  | |
| Rural | 0 |  | |
| Urban | -0.577 (-1.269, 0.115) | 0.1021 | |
| CCI Score |  |  | |
| 0 | 0 |  | |
| 1 | 0.276 (-0.130, 0.682) | 0.183 | |
| >=2 | -0.385 (-0.901, 0.132) | 0.1443 | |
| Diagnosis year | -0.029 (-0.068, 0.010) | 0.1489 | |
| **County-level characteristics** |  |  | |
| Population density | 0.000 (-0.002, 0.001) | 0.3701 | |
| % Male | 0.060 (-0.156, 0.277) | 0.5841 | |
| % Black | -0.009 (-0.029, 0.011) | 0.3752 | |
| % Poverty | -0.004 (-0.015, 0.006) | 0.4222 | |
| % Unemployed | -0.052 (-0.172, 0.068) | 0.3934 | |
| % No access to vehicle | 0.007 (-0.162, 0.175) | 0.9392 | |
| % Less than high school | 0.031 (-0.043, 0.105) | 0.4094 | |
| Ryan White centers | 0.032 (-0.072, 0.136) | 0.5431 | |
| Mental health provider | -0.077 (-0.157, 0.003) | 0.0589 | |
| Dissimilarity index | -1.934 (-5.222, 1.355) | 0.2492 | |

MSM: Men who have sex with men; IDU: Injection drug users; CCI: Charlson Comorbidity Index

# Supplemental SAS code: Generalized estimating equations

**Code 1: Generalized estimating equations model for binary outcome “Late diagnosis” using log-binomial link function to estimate the prevalence ratio. The estimated beta is the estimated log(PR). Thus, exp(beta) is the estimated PR.**

ods output GEEEmpPEst=est;

/*Note: no descending is probability of 0; descending is probability of 1*/

**proc** **gee** data=final1 descending;

class county agegp(ref="18-34") sex(ref="Female") race(ref="White") urban(ref="Rural")

risk(ref="Heterosexual") cci(ref="0")/PARAM=GLM;

model late= agegp sex race risk urban cci yr CEN_POPDENSITY_COUNTY ACS_PCT_MALE ACS_PCT_BLACK

ACS_TOT_CIVIL_POP_POV

ACS_PCT_UNEMPLOY ACS_PCT_HU_NO_VEH ACS_PCT_LT_HS prw1 pmh1 Dissimilarity/ dist=binomial link=log;

repeated subject=county / type=CS corrb;

**run**;

**data** est1;

set est;

keep Parm Level1 est p;

length est $30.;

if estimate=**0** and lowerCL=**0** and upperCL=**0** then do;

est="Ref";

end;

else do;

est0 = put(round(exp(estimate),**0.001**),**6.3**);

est1 = put(round(exp(lowerCL),**0.001**),**6.3**);

est2 = put(round(exp(upperCL),**0.001**),**6.3**);

est = catt(est0," (",est1,",",est2,")");

end;

length p $7.;

if probz = **.** then p=""; else if probz<**0.0001** then p = "<0.0001"; else p = round(probz,**.0001**);

**run**;

**proc** **print** data = est1 noobs;

**run**;

**Code 2: Generalized estimating equations model for continuous outcome “delay years” using identity link function.**

ods output GEEEmpPEst=est;

**proc** **gee** data=subset;

class county agegp(ref="18-34") sex(ref="Female") race(ref="White") urban(ref="Rural")

risk(ref="Heterosexual") cci(ref="0")/PARAM=GLM;

model delaynew1= agegp sex race risk urban cci yr CEN_POPDENSITY_COUNTY ACS_PCT_MALE ACS_PCT_BLACK

ACS_TOT_CIVIL_POP_POV

ACS_PCT_UNEMPLOY ACS_PCT_HU_NO_VEH ACS_PCT_LT_HS prw1 pmh1 Dissimilarity/ link=identity;

repeated subject=county / type=ar(**1**) corrb;

**run**;

**data** est1;

set est;

keep Parm Level1 est p;

length est $30.;

if estimate=**0** and lowerCL=**0** and upperCL=**0** then do;

est="Ref";

end;

else do;

est0 = put(round((estimate),**0.001**),**6.3**);

est1 = put(round((lowerCL),**0.001**),**6.3**);

est2 = put(round((upperCL),**0.001**),**6.3**);

est = catt(est0," (",est1,",",est2,")");

end;

length p $7.;

if probz = **.** then p=""; else if probz<**0.0001** then p = "<0.0001"; else p = round(probz,**.0001**);

**run**;

**proc** **print** data = est1 noobs;

**run**;
